# Two Novel Mosquitocidal Peptides Isolated from the Venom of the Bahia Scarlet Tarantula (*Lasiodora klugi*)

**DOI:** 10.3390/toxins15070418

**Published:** 2023-06-27

**Authors:** Jamila Ahmed, Andrew A. Walker, Hugo D. Perdomo, Shaodong Guo, Samantha A. Nixon, Irina Vetter, Hilary I. Okoh, Dalhatu M. Shehu, Mohammed N. Shuaibu, Iliya S. Ndams, Glenn F. King, Volker Herzig

**Affiliations:** 1 Department of Zoology, Ahmadu Bello University Zaria, Kaduna 810107, Nigeria; 2 Institute for Molecular Bioscience, The University of Queensland, Brisbane, QLD 4072, Australia; 3 Australian Research Council Centre of Excellence for Innovations in Peptide and Protein Science, University of Queensland, Brisbane, QLD 4072, Australia; 4 School of Biological Sciences, The University of Queensland, Brisbane, QLD 4072, Australia; 5 School of Pharmacy, The University of Queensland, Brisbane, QLD 4102, Australia; 6 Department of Animal and Environmental Biology, Federal University Oye-Ekiti, Oye 371104, Nigeria; 7 Department of Biochemistry, Ahmadu Bello University Zaria, Kaduna 810107, Nigeria; 8 Centre for Biotechnology Research and Training, Ahmadu Bello University Zaria, Kaduna 810107, Nigeria; 9 Centre for Bioinnovation, University of the Sunshine Coast, Sippy Downs, QLD 4556, Australia; 10 School of Science, Technology, and Engineering, University of the Sunshine Coast, Sippy Downs, QLD 4556, Australia

**Keywords:** *Lasiodora klugi*, insecticidal toxin, *Aedes aegypti*, disulfide-directed β-hairpin, inhibitor cystine knot

## Abstract

Effective control of diseases transmitted by *Aedes aegypti* is primarily achieved through vector control by chemical insecticides. However, the emergence of insecticide resistance in *A. aegypti* undermines current control efforts. Arachnid venoms are rich in toxins with activity against dipteran insects and we therefore employed a panel of 41 spider and 9 scorpion venoms to screen for mosquitocidal toxins. Using an assay-guided fractionation approach, we isolated two peptides from the venom of the tarantula *Lasiodora klugi* with activity against adult *A. aegypti.* The isolated peptides were named U-TRTX-Lk1a and U-TRTX-Lk2a and comprised 41 and 49 residues with monoisotopic masses of 4687.02 Da and 5718.88 Da, respectively. U-TRTX-Lk1a exhibited an LD_50_ of 38.3 pmol/g when injected into *A. aegypti* and its modeled structure conformed to the inhibitor cystine knot motif. U-TRTX-Lk2a has an LD_50_ of 45.4 pmol/g against adult *A. aegypti* and its predicted structure conforms to the disulfide-directed β-hairpin motif. These spider-venom peptides represent potential leads for the development of novel control agents for *A. aegypti*.

## 1. Introduction

Arboviral diseases like dengue, yellow fever, chikungunya, and zika are primarily vectored by the mosquito *Aedes aegypti* [1,2]. Dengue is the most important arboviral disease transmitted by *A. aegypti*, with an estimated 390 million annual cases and over half of the world population in 129 countries at risk of infection [2,3]. Yellow fever, an acute hemorrhagic viral disease, accounts for over 29,000 annual deaths with the disease being endemic in 47 countries [1]. Chikungunya is a mosquito-borne viral disease that has been identified in 42 countries in Africa, Asia, the Americas, and Europe [4]. Zika virus infections have been reported in Asia, Africa, the Pacific, and the Americas, with its infection being associated with microcephaly in Brazil [5].

Successful control of these diseases is mainly achieved through vector control using chemical insecticides. However, the emergence of resistance in *A. aegypti* to major insecticide classes such as pyrethroids, carbamates, and organophosphates threatens the control of these diseases [6,7]. Furthermore, chemical insecticides affect non-target beneficial organisms, cause environmental pollution, and threaten human health upon inhalation or accidental consumption. A safe alternative could be insect-selective toxins that have been patented for their possible use to control insect vectors [8].

Spiders are among the most successful and diverse venomous animals with an estimated 120,000 extant species [9], of which ~51,000 belonging to 132 families have been formally recognized [10]. Having evolved during the Ordovician period (450 million years ago), they are found in all types of habitats except for polar regions, the highest mountains, and the oceans [8]. Their success is partially attributed to the use of venom to rapidly subdue prey which mainly consists of a wide variety of other arthropods, including medically important disease vectors and agricultural pests [11,12,13]. Thus, spiders play an important ecological role in keeping insect populations at bay [14]. To enable spiders to overcome diverse types of prey, their venom comprises a complex mixture of active biological components, including proteins, peptides, acylpolyamines, small amines, histamine, and other small molecules [12,15,16,17]. Peptides are the most abundant components in their venom, with cysteine-rich peptides being the most important functional component [17]. Several insecticidal peptides have been isolated from the venom of various spider species and some have been patented as bio-insecticidal leads. One of these peptides has been developed commercially by Vestaron Corporation [8]. According to the ArachnoServer spider toxin database, over 230 spider-venom peptides have been reported to be insecticidal based on experimental data or predictions based on sequence homology [18]. These peptides act on a diverse range of molecular targets, including voltage-gated calcium channels, voltage-gated sodium channels, calcium-activated potassium channels, presynaptic nerve terminals, lipid bilayers, nicotinic acetylcholine receptors, and *N*-methyl-D-aspartate (NMDA) receptors [8,19,20,21].

Scorpions are the oldest group of arachnids, having evolved in the Silurian period [22,23]. To date, 2722 species have been described [24], and they are widely distributed and adapted to different terrestrial habitats except for Antarctica. Like spiders, their success is attributed to their use of venom for prey capture and defense against predators [25]. Their venoms are a complex mixture of bioactive compounds such as inorganic salts, amino acids, nucleic acids, peptides, mucopolysaccharides, and proteins [26,27]. Therefore, spider and scorpion venoms are considered rich repositories for the discovery of novel toxins to control insect pest and vectors [8,20,21,28,29,30,31].

Efficient delivery is considered the primary obstacle to deployment of venom-derived peptides as bio-control agents [32]. In 2007, the entomopathogenic fungus *Metarhizium anisopliae* was engineered to express AaIT, a toxin isolated from the venom of the scorpion *Androctonus australis*. The transgenic fungus was observed to reduce the kill time of *A. aegypti* as compared to the wild-type fungus [33]. Similarly, a pathogenic fungus (*Metarhizium pingshaense*) engineered with an insect-specific toxin from spider venom was reported to reduce the *Anopheles* mosquito population during a field trial in Burkina Faso by 90% [34]. Furthermore, in 2017 the US Environmental Protection Agency approved use of the SPEAR™ (Vestaron, Durham, NC, USA) range of bioinsecticides, in which the active component is a peptide derived from the venom of an Australian funnel-web spider [35]. To further expand the insecticidal armory against disease vectors such as mosquitoes, our study was designed to screen a diverse panel of 50 arachnid venoms with the aim of isolating and characterizing venom peptides that are active against adult *A. aegypti* mosquitoes.

## 2. Results

### 2.1. Isolation and Purification of Mosquitocidal Tarantula Venom Peptides

**Figure 1:**
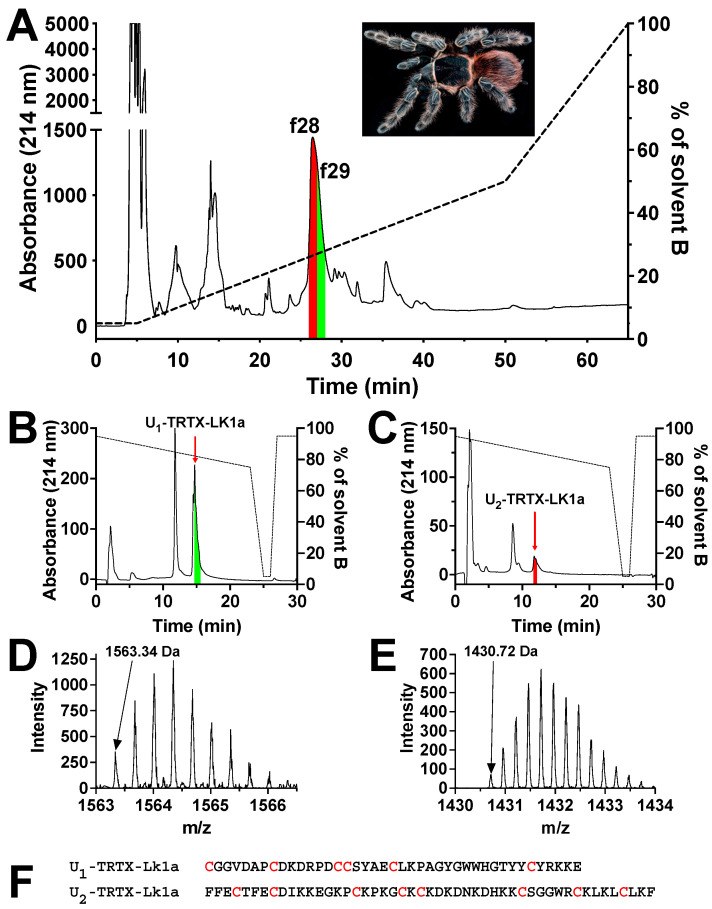
**Isolation and purification of mosquitocidal peptides.** (**A**) The crude venom of female *Lasiodora klugi* (pictured in the insert, photo courtesy of Bastian Rast) was fractionated using RP-HPLC and fractions 28 and 29 exhibited mosquitocidal activity against *A. aegypti*. (**B**,**C**) Further fractionation using HILIC-HPLC resulted in the purification of U-TRTX-Lk1a from fraction 28 and U-TRTX-Lk2a from fraction 29 (red arrows pointing to respective toxin peaks). (**D**,**E**) The molecular masses (black arrows pointing to the monoisotopic masses) were identified by electrospray mass spectrometry as 4687.02 Da for U-TRTX-Lk1a (based on the 3^+^ ion) and 5718.88 Da for U-TRTX-Lk2a (based on the 4^+^ ion). (**F**) Peptide sequences for U-TRTX-Lk1a and U-TRTX-Lk2a as determined by Edman degradation and de novo sequencing. Cysteine residues are highlighted in red.

We performed a preliminary insecticidal screen of 41 spider and 9 scorpion venoms by injecting 6.25 ng of each venom into five adult *A. aegypti* mosquitoes (Supplementary Tables S1 and S2). Potent mortality of >50% was observed in 19 venoms (=38%) at 24 h post-injection with 12 tarantula venoms causing 80–100% mortality (Supplementary Figure S1), while none of the scorpion venoms caused mortality above 20% (Supplementary Table S2). The venom of *L. klugi* caused 100% irreversible paralysis at 0.5 h post injection leading to over 90% mortality after 2 h. Bioassay-guided fractionation of *L. klugi* venom using a C_18_ reversed-phase (RP) HPLC column revealed that the fractions eluting from 26 to 28 min (Figure 1A) caused irreversible contractile paralysis in 100% of injected mosquitoes and ultimately death after 24 h. These fractions were sub-fractionated using a hydrophilic interaction liquid chromatography (HILIC) column (Figure 1B,C), which resulted in the isolation of two active peptides which eluted at ~12 min and 15 min, and were named U-TRTX-Lk1a (Lk1a) and U-TRTX-Lk2a (Lk2a) according to the rational nomenclature for peptide toxins [36]. Mass spectrometric analysis revealed the monoisotopic mass to be 4687.02 Da for Lk1a and 5718.88 Da for Lk2a (Figure 1D,E).

### 2.2. Mosquitocidal ACTIVITY

**Figure 2:**
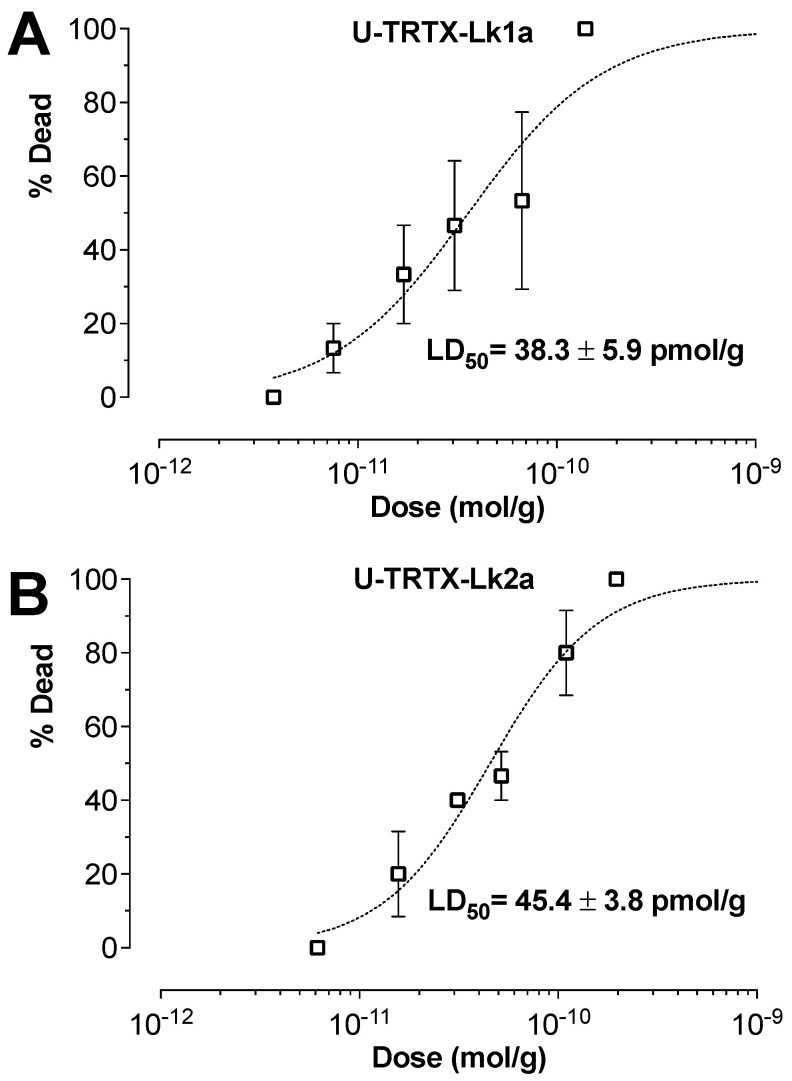
**Mosquitocidal activity.** Dose–response curves for the toxicity of the venom peptides Lk1a (**A**) and Lk2a (**B**) as observed 24 h after injection into adult *Aedes aegypti*.

Upon injection into *A. aegypti*, both toxins caused irreversible contractile paralysis resulting in 100% mortality after 24 h. Lk1a had an LD_50_ of 38.3 pmol/g (Figure 2A), while Lk2a had an LD_50_ of 45.4 pmol/g after 24 h (Figure 2B).

### 2.3. Primary Structure Determination for Mosquitocidal Venom Peptides

Using a combination of Edman sequencing and de novo liquid chromatography–tandem mass spectrometry (LC-MS/MS), complete peptide sequences were determined for both Lk1a and Lk2a. For Lk1a, Edman sequencing at the Australian Proteome Analysis Facility returned the partial N-terminal sequence CGGVDAPCDKKRPDCCS(S)AECLK(P)AG-(G), with brackets indicating a tentative assignment. This sequence closely matches the previously reported peptide U_2_-TRTX-Lsp1a CGGVDAPCDKDRPDCCSSAECLKPAGYGWWHGTYYCYRKRER from *Lasiodora* sp. (https://arachnoserver.qfab.org/toxincard.html?id=509, accessed 27 March 2022). Manual analysis of LC-MS/MS data from reduced, alkylated, and trypsinized Lk1a revealed that the isolated peptide ends in TYYCYRKKE (carboxyl terminus), indicating a conservative R41K polymorphism compared to the database peptide, with the final R residue likely removed by carboxypeptidase similar to other spider-venom peptides [12]. Examination of matrix-assisted laser desorption/ionization (MALDI-TOF) MS spectra with fragmentation induced by 1,5-diaminonaphthalene (1,5-DAN) yielded the internal fragmentary sequence DCCSYAE, indicating Y rather than the S that was tentatively called by Edman sequencing at the 18th residue. Taken together, the combined data yields a putative sequence of CGGVDAPCDKDRPDCCSYAECLKPAGYGWWHGTYYCYRKKE with a predicted monoisotopic mass of 4686.973 Da, which closely matched the measured monoisotopic mass of this peptide (4687.02 Da).

For Lk2a, manual de novo peptide sequencing from LC-MS/MS data yielded the tryptic fragment CSGGWR. A search of the ArachnoServer database [18] with this fragment returned the peptide U_1_-TRTX-Lsp1a FFECTFECDIKKEGKPCKPKGCKCKDKDNKDHKKCSGGWRCKLKLCLKF from *Lasiodora* sp. venom (https://arachnoserver.qfab.org/toxincard.html?id=669, accessed 27 March 2022) with a predicted monoisotopic mass of 5718.80 Da, which closely matches the measured monoisotopic mass of the native toxin, 5718.88 Da.

**Figure 3:**
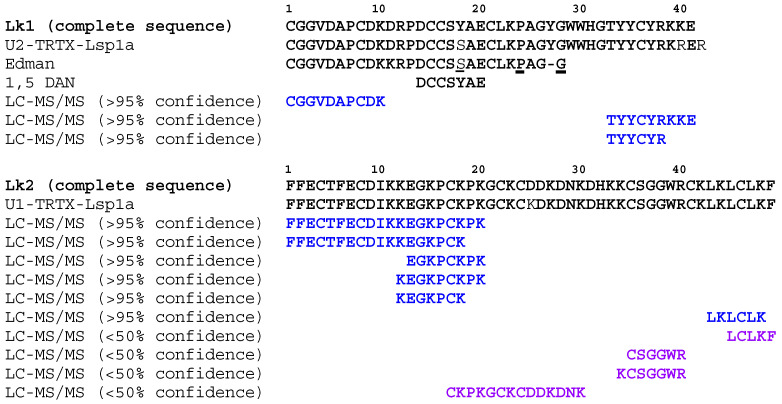
**Primary structure determination.** The primary structure of Lk1 and Lk2 was determined using a combination of N-terminal Edman sequencing, LC-MS/MS analysis, 1,5-diaminonaphthalene induced fragmentation, and homology searches in public databases. All residues matching with the complete sequence are indicated in bold, tentative calls from Edman are underlined, the colors indicate different confidence levels for the de novo LC-MS/MS sequencing (blue > 95% confidence; purple < 50% confidence). Residue numbers are shown above the sequences.

To further examine the mass spectral evidence for these peptide primary structures, we compared the LC-MS/MS data of reduced, alkylated, and trypsinized peptides with a sequence database containing the putative Lk1a and Lk2a sequences, all amino acid sequences from Arachnida on UniProt, and 200 common MS contaminants. Lk1a and Lk2a were the top detected peptides in each sample. Three tryptic peptides originating from Lk1a were confidently detected, covering 49% of the sequence. Five tryptic peptides originating from Lk2a were detected, covering 53% of the sequence, including the 20 N-terminal residues of the peptide. Together, the MS data provides good support for the determined primary structures of these peptides (Figure 3).

**Figure 4:**
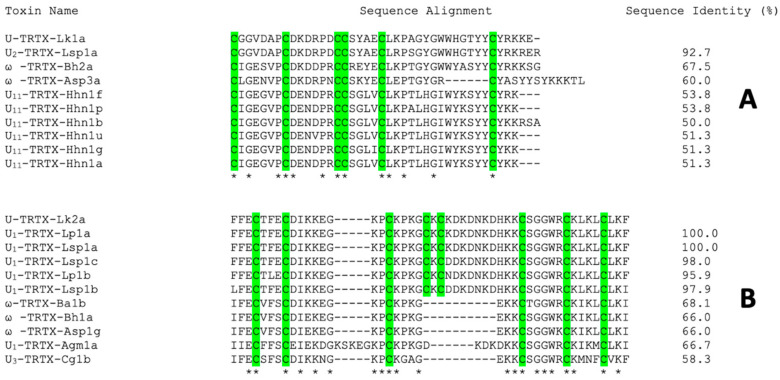
**Multiple sequence alignments.** The closest matching spider-venom peptide sequences for (**A**) Lk1a and (**B**) Lk2a. Homologous sequences were identified by BLAST searches of the NCBI and ArachnoServer) databases, and the resulting sequences were aligned using ClustalX (version 2.0). Cysteine residues are highlighted in green and asterisks indicate conserved regions in the alignment. The percent sequence identity to the mosquitocidal toxins from *L. klugi* is indicated on the right.

Basic local alignment searches of amino acid sequences of isolated peptides revealed several peptides from tarantula venoms with sequence identities of 51–100%. There are 16 and 21 conserved sites in the multiple sequence alignments of Lk1a and Lk2a, respectively (Figure 4). All toxins with characterized molecular targets in both alignments are modulators of voltage-gated calcium (Ca_V_) channels.

### 2.4. In Silico Structures of Isolated Peptides

**Figure 5:**
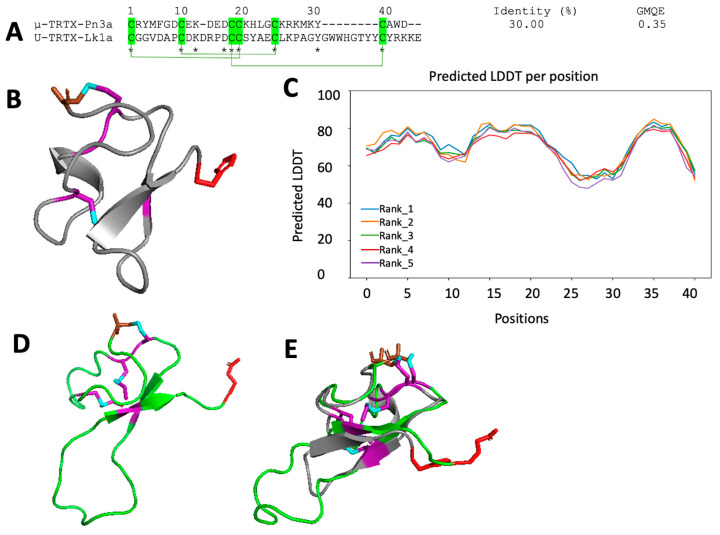
**Predicted 3D structure of Lk1a.** (**A**) Multiple sequence alignment of Lk1a with its template μ-TRTX-Pn3a; GMQE, global model quality estimation; *, conserved site; (**B**) predicted structure of Lk1a using SwissModel; (**C**) predicted local distance difference test per residue position using AlphaFold2; (**D**) predicted structure of Lk1a using AlphaFold2; (**E**) overlap of SwissModel- and AlphaFold2-predicted structures of Lk1a. The cysteine regions are highlighted in green/purple. The N-terminal and C-terminal of all the structures are indicated in brown and red, respectively.

Lk1a is comprised of 41 amino acid residues, including 6 cysteines, while Lk2a is comprised of 49 amino acids, including 8 cysteines. SWISS-MODEL (https://swissmodel.expasy.org, accessed 17 November 2022) and AlphaFold2 Colab (https://colab.research.google.com/github/sokrypton/ColabFold/blob/main/AlphaFold2.ipynb, accessed 17 November 2022) was used to predict the structures of the mosquitocidal peptides. Using SWISS-MODEL, the 30-residue peptide μ-TRTX-Pn3a (PDB code: 5T4R) from venom of the tarantula *Pamphobeteus nigricolor* [37] was used as a template to model Lk1a. Sequence alignment revealed 30% sequence identity and 0.35 global model quality estimation (GMQE) (Figure 5A). The two peptides have a similar cysteine framework with few conserved sites. The modeled structure conforms to the inhibitor cystine knot (ICK) motif [38] having two antiparallel β-sheets (E20–C22 and G26–W29) connected by a loop and a small α-helix (R12-D14) (Figure 5B). Similarly, the structure predicted by AlphaFold2 conforms to the ICK motif (Figure 5D) having a predicted local distance difference test of 60–85% (Figure 5C). Both modeled structures have three disulfide bonds: C1–C16, C8–C21, and C15–C36 (Figure 5A). When the two predicted structures were superimposed, the major difference was a string of residues connecting the two β sheets that are longer in the structure predicted by AlphaFold2 (Figure 5E). Importantly, the structure predicted by AphaFold2 is made up of all 41 residues, while the predicted SWISS-MODEL structure is missing the last 10 residues (Figure 5B,D). Additionally, the structure predicted by SWISS-MODEL has a small α helix (Figure 5B).

**Figure 6:**
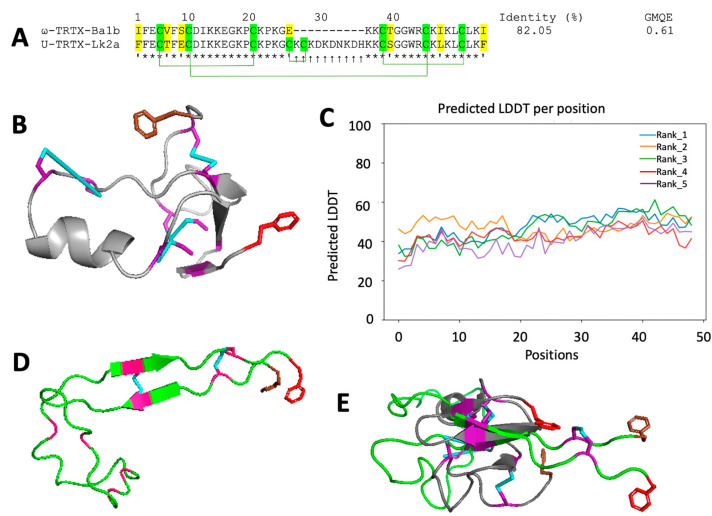
**Predicted 3D structure of Lk2a.** (**A**) Multiple sequence alignment of Lk2a with its template ω-TRTX-Ba1b; GMQE, global model quality estimation; *, conserved sites; ↑, insertion; ‘, mutations; (**B**) predicted structure of Lk2a using SwissModel; (**C**) predicted local distance difference test per residue position using AlphaFold2; (**D**) predicted structure of Lk2a using AlphaFold2; (**E**) aligned predicted structures of Lk2a. The cysteine regions are highlighted in green/purple. The N-terminal and C-terminal of all the structures are indicated in brown and red, respectively.

The structure of the Lk2a peptide was predicted using ω-TRTX-Br1b (PDB code: 2KGH) from *Brachypelma albiceps* (previously *B. ruhnaui*) as a template [39]. The peptide and template have 82% sequence identity and 0.61 GMQE. The sequences have a similar cysteine framework; however, Lk2a contains eight cysteines, and was predicted to have four disulfide bonds, while ω-TRTX-Ba1b contains six cysteines, forming three disulfide bonds. The sequences also differ by seven residues (indicated in yellow in Figure 6A), with a large insertion between positions 22 and 31 in Lk2a (Figure 6A). The predicted 3D structure of Lk2a conforms to the disulfide-directed β-hairpin motif [40] having three anti-parallel β-sheets stabilized by three disulfide bonds. Interestingly, this structure also contains an α-helix and a fourth disulfide bond. The first β-sheet (K15–C17) is separated from the second β-sheet (G38–K41) by an α-helix (D32–K33) while the second β-sheet is connected to the third (L45–L47) by a loop (Figure 6B). The Lk2a structure predicted by AlphaFold2 (Figure 6D) has a very low predicted local distance different test (PLDDT, Figure 6C), and as such does not give a reliable picture of the structure. None of the predicted regions matched when both structures were overlayed (Figure 6E). Experimental analysis using NMR spectroscopy will be required in the future to verify the predicted fold of Lk2a.

## 3. Discussion

*Aedes aegypti* is a vector of several viral diseases that have resulted in a substantial economic burden and the loss of many human lives [1,2,3,4,5]. Several control measures have been put in place to control diseases vectored by this mosquito, which are centered on vector control achieved mainly using chemical insecticides. However, the development of resistance to chemical insecticides by *Aedes aegypti* [6,7] poses a threat to this control method. As such, there is an urgent need to develop alternative control methods. To aid the development of eco-friendly biocontrol methods, we screened 50 arachnid venoms for insecticidal activity against *A. aegypti,* and potent insecticidal activity was observed in the venom of Bahia scarlet tarantula *L. klugi*. Using a combination of RP-HPLC and HILIC, two potent insecticidal peptides, namely, Lk1a and Lk2a, were isolated from the venom of *L. klugi*. These peptides caused irreversible paralysis and eventually mortality in *A. aegypti* adults but differed slightly in their potency. To the best of our knowledge, these are the first insecticidal peptides isolated by directly screening for activity against *A. aegypti* mosquitoes. However, other theraphosid venom peptides with insecticidal activity against dipterans have been reported, e.g., Ae1a causing irreversible paralysis in *Drosophila melanogaster* [41] and two venom peptides from *Monocentropus balfouri* causing paralysis in *Lucilia cuprina* and *Musca domestica* [42]. An important question that needs to be addressed in future studies is how the two mosquitocidal leads Lk1a and Lk2a might be delivered for controlling mosquitoes in the field. An approach that has already produced promising results is the use of entomopathogens for toxin delivery [43,44]. Entomopathogens like *Isaria fumosorosea* have been reported to be potent against *A. aegypti* [45] and could therefore be engineered to produce Lk1a and Lk2a. This method of deployment offers several advantages like reduction in kill time, improved oral activity, and increased phyletic specificity [46]. A field trial in Burkina Faso using an entomopathogenic fungus engineered to express an insecticidal spider toxin (ω/κ-hexatoxin) demonstrated that spider-venom peptides can be successfully employed to control *Anopheles* populations under field conditions [34].

The sequences of the isolated mosquitocidal peptides were elucidated using Edman degradation and LC-MS/MS analysis. Lk1a and Lk2a contain 41 and 49 residues, with a monoisotopic mass of 4687.02 Da and 5718.88 Da, respectively (Figure 1). Both peptides contain cysteine residues but differ in their disulfide architecture. Several anti-insect toxins have been reported to contain cysteine residues that form disulfide bonds [8]. Furthermore, a search of public databases using the Lk1a and Lk2a sequences revealed similarity with anti-insect toxins from *Brachypelma harmorii* (ω-TRTX-Bh2a and ω-TRTX-Bh1a) and *Brachypelma albiceps* (ω-TRTX-Ba1b). These peptides have been reported to be toxic to crickets by inhibiting Ca_V_ channels, but not toxic to mice [39], suggesting they might be good insecticidal leads. Given the similarity with ω toxins, it seems obvious to suggest that the two toxins isolated in this study are likely to be Ca_V_ channel modulators.

The 3D structures of the two mosquitocidal toxins were predicted using SWISS-MODEL and AphaFold2. Two structural motifs were predicted: the ICK, or knottin, motif and the disulfide-directed β-hairpin (DDH). The ICK and DDH motifs are the most common, with ICK peptides accounting for more than 90% of toxins in some spider venoms [42]. The predicted Lk1a structure using AlphaFold2 Colab and SWISS-MODEL conforms with the ICK motif (Figure 5B,D). This motif is characterized by a cystine knot [38] which is potentially advantageous for insecticidal leads due to the inherently high stability of ICK peptides [8,47,48,49]. The predicted Lk2a structure using SWISS-MODEL (Figure 6B) is an elaborated DDH motif having an α-helix and four disulfide bonds. Such elaborations in the disulfide architecture have been reported to play an important role in peptide diversification [47], and several studies have reported the DDH motif from spider and scorpion venom peptides [37,50,51,52]. The Lk2a structure predicted using AlphaFold2 Colab does not conform with any known venom peptide structure. Due to the low PLDDT (<50), which is a measure of confidence, further experimental evidence, for example, using NMR spectroscopy, is required to determine its 3D structure and cysteine connectivity.

## 4. Conclusions

We discovered two mosquitocidal peptides Lk1 and Lk2 from the venom of the Bahia scarlet tarantula *Lasiodora klugi*. Further experiments are required to determine the best strategies for applying these leads under field conditions for controlling *Aedes aegypti* mosquitoes and consequently the diseases they transmit.

## 5. Materials and Methods

### 5.1. Rearing of Aedes aegypti

Mosquitoes were reared according to the method of Perdomo et al. [53]. Plastic cups were filled (1/3) with deionized water. Filter papers were submerged in the cups, which were kept in cages containing adult mosquitoes. Glass plate artificial feeders using parafilm membrane were used to blood feed female adult mosquitoes. Blood (containing acid citrate dextrose as an anti-coagulant) was obtained from Australian Red Cross Services (Kelvin Grove, QLD, Australia) and maintained at a temperature of 37 °C by circulating water through the feeders. Following the laying of eggs on the filter papers, each filter paper containing *Aedes aegypti* eggs was placed in a flat plastic tray filled with distilled water and grounded fish feed was added. This setup was observed after 24 h for hatched larvae and monitored every day for pupae. Pupae were transferred into fresh plastic cups which were placed in adult cages. The emerged adults were fed 5% sucrose and maintained at 27 ± 1 °C temperature, relative humidity of 75–80%, and 12 h alternating photoperiods.

### 5.2. Venom Extraction

Venoms were sourced from 41 spider and 9 scorpion species. Spider venoms were collected by electrical stimulation of the basal part of the chelicerae [54] while scorpion venoms were collected by forcing aggravated scorpions to sting a sheet of parafilm, from where the venom was then collected [13]. After collection, the venoms were lyophilized, and venom stock solutions were prepared by reconstituting with Milli-Q water.

### 5.3. Mosquito Toxicity Bioassay

For each venom, five adult female mosquitoes with an average weight of 3.19 mg were anesthetized on ice and with the aid of a nanoinjector (Nanoject III, Drummond Scientific Company, Broomall, PA, USA) and a binocular dissecting microscope (Nikon SMZ800, Nikon Instrument Inc., Melville, NY, USA) and 6.25 ng of venom reconstituted in phosphate buffer saline (PBS) was then injected into the ventrolateral thoracic region. The mosquitoes were placed in transparent plastic cups and observed after 0.5, 1, 2, and 24 h for paralysis or death [13]. This experiment was replicated thrice, and mosquitoes injected with PBS only served as the negative control. Using a similar injection procedure, two purified active *L. klugi* peptides were subjected to adulticidal toxicity assays. These consisted of injecting 6 doses between 0.06 and 4 ng of each peptide into N = 5 mosquitoes in triplicate. An additional N = 5 mosquitoes for each dose were injected with PBS as a control. The resulting LD_50_ was determined using a sigmoidal dose–response curve (variable slope) in Prism 8 (Graphpad Software, San Diego, CA, USA) as previously described [13].

### 5.4. Peptide Isolation

Bioassay-guided fractionation was used to isolate active peptides from *L. klugi* venoms by combining RP-HPLC and HILIC chromatography. For RP-HPLC, we used solvents A (0.09% formic acid (FA) in water) and solvent B (0.09% FA in 90% acetonitrile (ACN)). The equivalent of one milligram (based on the dried weight) of crude venom was dissolved in 5% solvent B (450 μL) and subjected to fractionation using a C_18_ Phenomenex Jupiter RP-HPLC column (250 × 4 mm, 5 μm, Phenomenex Jupiter, Sydney, Australia). Peptides were eluted at a flow rate of 0.7 mL/min (using the following gradient: 0–5 min: 5% B; 5–50 min: 5–50% B; 50–65 min: 50–100% B) and UV absorption was monitored at 214 nm [55]. The resulting venom fractions were injected into mosquitoes (see Section 5.3) and the active fractions were further subjected to HILIC-HPLC according to the method of Badgett et al. [56] using HILIC solvents A (trifluoroacetic acid (TFA) 0.5% in water) and B (90% ACN in 0.043 TFA). The venom fractions were dissolved in 450 μL of 95% HILIC solvent B and subfractions were eluted at a flow rate of 1 mL/min using the following gradient: 0–23 min: 95% B; 23–25 min: 75% B; 25–27 min: 5% B.

### 5.5. Proteomics

The purity and mass of isolated peptides were determined using MALDI-TOF mass spectrometry on an AB Sciex TOF/TOF 5800 (Framingham, MA USA) proteomic analyzer. Toxin samples were mixed 1:1 (*v*:*v*) with α-cyano-4-hydroxy-cinnamic acid matrix (6 mg/mL in 50/50 acetonitrile/H_2_O with 5% FA) and MALDI-TOF spectra were acquired in reflector positive mode. 

Similarly, 1,5-diaminonaphthalene (1,5-DAN) was used to induce fragmentation in Lk1a by mixing it with the toxin in a 1:1 (*v*:*v*) ratio. MALDI-TOF spectra for prepared samples were acquired using reflector positive mode.

For LC-MS/MS, native untreated peptides were prepared by diluting fractionated peptides to 25 µL in 1% FA. The method of Walker et al. [57] was used to prepare reduced, alkylated, and trypsinized peptides for de novo sequencing. Peptide samples (4 μg) were incubated in 40 μL of reducing alkylating agent (4.875 mL ACN, 4.5 mL Milli-Q water, 0.5 mL 1 M ammonium carbonate pH 11.0, 100 μL 2-iodoethanol, 25 μL triethylphosphine) for 37 °C for 1 h before drying. Peptides were then resuspended in 10 μL trypsin reagent (40 ng/μL of trypsin in 50 mM ammonium bicarbonate pH 8.0 and 10% ACN). An extraction agent (50% ACN, 5% FA) was added to inactivate the trypsin, and prepared samples were dried and resuspended in 40 μL of 1% FA.

Peptide samples were loaded onto a Zorbax 300SB-C18 column (Agilent #858750-902) on a Shimadzu Nexera X2 LC system, and eluted using a 14 min gradient of 1–40% solvent B (90% ACN/0.1% FA) in solvent A (0.1% FA) at a flow rate of 0.2 mL/min. The LC outflow was coupled to a 5600 Triple TOF mass spectrometer (SCIEX) equipped with a Turbo V ion source. For MS1 scans, *m*/*z* was set between 350 and 2200. Precursor ions with *m*/*z* 350–1500, charge of +2 to +5, and signals with >100 counts/s (excluding isotopes within 2 Da) were selected for fragmentation, and MS2 scans were collected over a range of 80–1500 *m*/*z*. The resulting MS/MS spectra data were analyzed using PEAKS^®^ studio version 5.2 software (Bioinformatics Solutions Inc., Waterloo, ON, Canada) for de novo sequencing. Peptide masses were calculated using online software (https://www.peptidesynthetics.co.uk/tools/, accessed 27 March 2020).

### 5.6. Edman Sequencing

The first 26 residues of Lk1a were determined using Edman degradation and the remaining part of the sequence was determined using de novo LC-MS/MS sequencing while the sequence of Lk2a was determined using de novo LC-MS/MS only. N-terminal Edman sequencing was conducted by the Australian Proteome Analysis Facility (Sydney, NSW, Australia). Briefly, the peptide sample was solubilized in 25 mM ammonium bicarbonate/10% ACN, reduced using dithiothreitol (25 mM) at 56 °C for 0.5 h) and then alkylated using iodoacetamide (55 mM) at room temperature for 0.5 h. The prepared sample was then desalted/purified by RP-HPLC using a Zorbax 300SB-C18 column (3 × 150 mm), loaded onto a precycled, Biobrene-treated disc, and subjected to sequencing on an automated Applied Biosystems 494 Procise Protein Sequencing System [58].

The isolated peptides were named Lk1a and Lk2a according to King et al. [36]. ProtParam (http://web.expasy.org/protparam, accessed 25 March 2020) was used to compute parameters such as molecular weight, theoretical pI, and extinction coefficient. The resulting sequences were searched against a public database containing peptides from spiders (https://arachnoserver.qfab.org/mainMenu.html, accessed 27 March 2020) and the NCBI non-redundant (nr) database (https://blast.ncbi.nlm.nih.gov/Blast.cgi, accessed 27 March 2020) using the BLASTp algorithm with the expected value (e-value) cutoff set to <10^−5^ to determine similar sequences that are homologs of the insecticidal peptides [18,59]. Multiple sequence alignments were performed with CLUSTALX v2.0.

### 5.7. Structure Modeling

The structures of active peptides were predicted using SWISS-MODEL) [60] and AphaFold2 [61] Colab [62]. Templates were searched using target sequence with the aid of BLAST [63] and HHBlits [64] against the SWISS-MODEL template library (SMTL). Global model quality estimation (GMQE) was used to estimate the template-target quality. The models were built following target-template alignment using ProMod3 and PROMOD-II [65]. All templates used in this study were NMR solution structures that do not have ligands. Peptide sequences were copied into the query input section and run on the AlphaFold2 Colab (https://colab.research.google.com/github/sokrypton/ColabFold/blob/main/AlphaFold2.ipynb, accessed 17 November 2022) without a template. The resulting structural models were downloaded and edited using PyMOL v2.4.

## Data Availability

Not applicable.

## Supplementary Materials

The following supporting information can be downloaded at: https://www.mdpi.com/article/10.3390/toxins15070418/s1, Figure S1: Mean adulticidal activities of spider and scorpion venoms against adult *Aedes aegypti* after 24 hours of observation; Table S1: Adulticidal activity expressed in percentage of mortality of venoms isolated from spider species against adult *Aedes aegypti*. Spider taxonomy according to the World Spider catalog (https://wsc.nmbe.ch/), version 24, accessed 11 April 2023; Table S2: Adulticidal activity expressed in percentage of mortality of venoms isolated from scorpion species against adult *Aedes aegypti*. Scorpion taxonomy according to The Scorpion Files (https://www.ntnu.no/ub/scorpion-files/index.php), accessed 11 April 2023.
