# Supplementary material for: Two Novel Mosquitocidal Peptides Isolated from the Venom of the Bahia Scarlet Tarantula (Lasiodora klugi)
Source: Toxins (Basel). 2023 Jun 27;15(7):418. doi: 10.3390/toxins15070418 (PMC10467143; doi:10.3390/toxins15070418)
Supplement: Supplementary file 1 [file toxins-15-00418-s001.zip › toxins-2406401-supplementary.pdf]

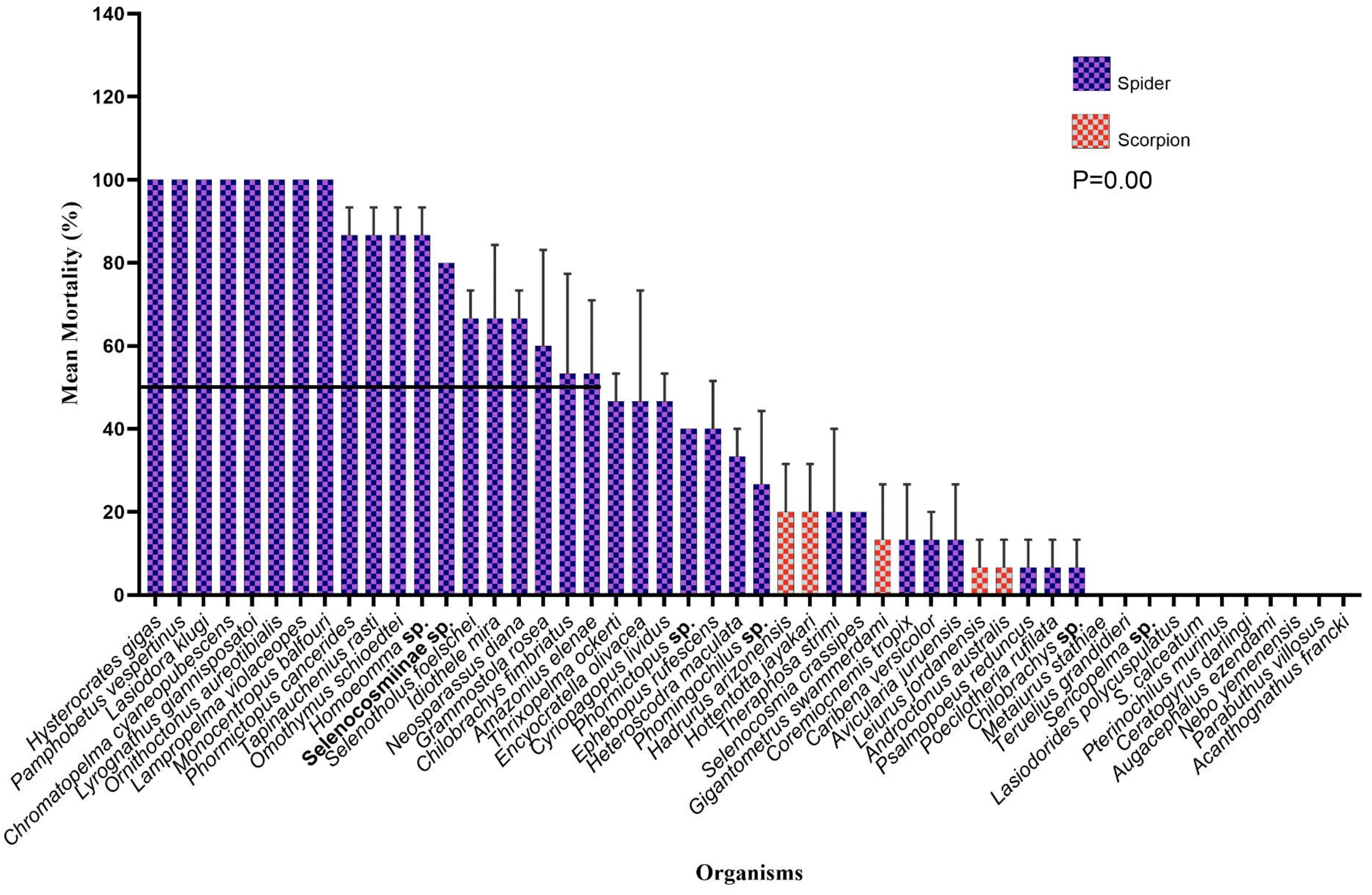

Figure S1: Mean adulticidal activities of spider and scorpion venoms against adult *Aedes aegypti* after 24 hours of observation

Table S1: Adulticidal activity expressed in percentage of mortality of venoms isolated from spider species against adult *Aedes aegypti*. Spider taxonomy according to the World Spider catalog (https://wsc.nmbe.ch/), version 24, accessed 11 April 2023.

		Paralysis and mortality with time {6.25ng/mosquito; 100 nl (62.5 ng/µl)}							
		30 min		1 h		2 h		24 h	
Family	Species	P	M	P	M	P	M	P	M
Nemesiidae	*Acanthogonatus francki*	6.7	0	6.7	0	6.7	0	0	0
Sparassidae	*Neosparassus diana*	60	0	60	0	60	0	66.7	66.7
Theraphosidae	*Amazonius elenae*	100	0	80	0	80	0	60	60
	Augacephalus ezendami	100	0	100	0	100	0	40	40
	Avicularia juruensis	100	0	100	0	100	0	100	13.3
	Caribena versicolor	100	0	86.7	0	86.7	0	33.3	13.3
	Ceratogyrus darlingi	86.7	0	86.7	0	86.7	0	0	0
	Chilobrachys sp.	93.3	0	93.3	0	93.3	0	40	6.7
	Chilobrachys fimbriatus	100	0	100	40	100	40	80	53.3
	Chromatopelma cyaneopubescens	100	0	100	6.67	100	80	100	100
	Coremiocnemis tropix	100	0	100	6.67	100	13.33	66.7	13.3
	Cyriopagopus lividus	80	0	80	0	0	0	66.7	46.7
	Encyocratella olivacea	40	0	100	0	100	0	80	46.7
	Ephebopus rufescens	100	0	100	0	100	0	80	40
	Grammostola rosea	100	0	100	0	100	0	80	60
	Heteroscodra maculata	80	0	86.7	0	86.7	0	33.3	33.3

P- paralysis; M- mortality; paralysis and mortality were not observed in mosquitoes injected with phosphate buffered saline

Table S1 continued: Adulticidal activity expressed in percentage of mortality of venoms isolated from spider species against adult *Aedes aegypti*. Spider taxonomy according to the World Spider catalog (https://wsc.nmbe.ch/), version 24, accessed 11.4.2023.

		Paralysis and mortality with time {6.25ng/mosquito; 100 nl (62.5 ng/µl)}							
		30 min		1 h		2 h		24 h	
Family	Species	P	M	P	M	P	M	P	M
Theraphosidae	*Homoeomma sp.*	100	0	100	0	100	0	100	86.7
	Hysterocrates gigas	100	0	100	0	100	20	100	100
	Idiothele mira	53.3	0	80	0	80	0	66.7	66.7
	Lasiodora klugi	100	20	100	60	100	93.3	100	100
	Lasiodorides polycuspulatus	0	0	46.7	0	60	0	6.7	0
	Lyrognathus giannisposatoi	100	20	100	80	100	100	100	100
	Monocentropus balfouri	100	0	100	0	100	0	100	0
	Omothymus schioedti	100	0	100	0	100	40	86.7	86.7
	Omothymus violaceopes	100	0	100	0	100	66.7	100	100
	Ornithoctonus aureotibialis	86.7	0	100	0	100	20	100	100
	Pamphobeteus vespertinus	100	0	100	0	100	13.3	100	100
	Phormictopus cancerides	100	0	100	0	100	13.3	100	86.6
	Phormictopus sp.	100	0	100	0	100	0	66.7	40
	Phormingochilus sp.	100	0	100	0	100	0	60	26.7
	Poecilotheria rufilata	100	0	100	0	86.7	0	20	6.7
	Psalmopoeus reduncus	46.7	0	86.7	0	86.7	6.7	6.7	6.7

P- paralysis; M- mortality; paralysis and mortality were not observed in mosquitoes injected with phosphate buffered saline

Table S1 continued: Adulticidal activity expressed in percentage of mortality of venoms isolated from spider species against adult *Aedes aegypti*. Spider taxonomy according to the World Spider catalog (https://wsc.nmbe.ch/), version 24, accessed 11.4.2023.

		Paralysis and mortality with time {6.25ng/mosquito; 100 nl (62.5 ng/µl)}							
		30 min		1 h		2 h		24 h	
Family	Species	P	M	P	M	P	M	P	M
Theraphosidae	*Pterinochilus murinus*	100	0	100	0	100	0	0	0
	Selenocosmia crassipes	100	0	100	6.7	100	6.7	20	6.7
	Selenocosmiinae sp.	100	0	13.3	0	13.33	6.7	80	80
	Selenotholus foelschei	100	0	100	46.7	100	66.7	100	66.7
	Sericopelma sp.	100	0	0	0	0	0	0	0
	Stromatopelma calceatum	100	0	0	0	0	0	0	0
	Tapinauchenius rasti	100	0	100	20	100	26.7	86.7	86.7
	Theraphosa stirmi	100	0	100	20	100	20	100	20
	Thrixopelma ockerti	100	0	100	0	100	0	80	46.7

P- paralysis; M- mortality; paralysis and mortality were not observed in mosquitoes injected with phosphate buffered saline

Table S2: Adulticidal activity expressed in percentage of mortality of venoms isolated from scorpion species against adult *Aedes aegypti*. Scorpion taxonomy according to The Scorpion Files (https://www.ntnu.no/ub/scorpion-files/index.php), accessed 11 April 2023.

		% Paralysis and Mortality with time {6.25ng/mosquito; 100 nl (62.5 ng/μl)}							
		0.5 h		1 h		2 h		24 h	
Family	Species	P	M	P	M	P	M	P	M
Buthidae	*Androctonus australis*	40	0	40	6.7	20	6.7	6.7	6.7
	Hottentotta jayakari	100	0	100	0	100	13.3	100	20
	Leiurus jordenensis	100	0	100	0	100	0	46.7	6.7
	Parabuthus villosus	0	0	0	0	0	0	0	0
	Teruelius grandidieri	26.7	0	26.7	0	20	0	0	0
Diplocentridae	*Nebo yemenensis*	0	0	0	0	0	0	0	0
Hadruridae	*Hadrurus arizonensis*	0	0	0	0	0	0	20	20
Iuridae	*Metaiurus stathiae*	0	0	0	0	0	0	0	0
Scorpionidae	*Gigantometrus swammerdami*	20	0	20	0	80	0	13.3	13.3

P- paralysis; M- mortality; paralysis and mortality were not observed in mosquitoes injected with phosphate buffered saline
